# Ghrelin peptide improves glial conditioned medium effects on neuronal differentiation of human adipose mesenchymal stem cells

**DOI:** 10.1007/s00418-021-01980-3

**Published:** 2021-03-16

**Authors:** Cristina Russo, Giuliana Mannino, Martina Patanè, Nunziatina Laura Parrinello, Rosalia Pellitteri, Stefania Stanzani, Rosario Giuffrida, Debora Lo Furno, Antonella Russo

**Affiliations:** 1 Physiology Section, Department of Biomedical and Biotechnological Sciences, University of Catania, via S. Sofia, 97, 95123 Catania, Italy; 2 Division of Hematology, AOU “Policlinico-San Marco”, via S. Sofia, 78, 95123 Catania, Italy; 3 Institute for Biomedical Research and Innovation, Italian National Research Council, Via P. Gaifami, 18, 95126 Catania, Italy

**Keywords:** Human adipose mesenchymal stem cells, Ghrelin, Neural-like differentiation, Glial conditioned media, Olfactory ensheathing cells, Schwann cells

## Abstract

The influences of ghrelin on neural differentiation of adipose-derived mesenchymal stem cells (ASCs) were investigated in this study. The expression of typical neuronal markers, such as protein gene product 9.5 (PGP9.5) and Microtubule Associated Protein 2 (MAP2), as well as glial Fibrillary Acid Protein (GFAP) as a glial marker was evaluated in ASCs in different conditions. In particular, 2 µM ghrelin was added to control ASCs and to ASCs undergoing neural differentiation. For this purpose, ASCs were cultured in Conditioned Media obtained from Olfactory Ensheathing cells (OEC-CM) or from Schwann cells (SC-CM). Data on marker expression were gathered after 1 and 7 days of culture by fluorescence immunocytochemistry and flow cytometry. Results show that only weak effects were induced by the addition of only ghrelin. Instead, dynamic ghrelin-induced modifications were detected on the increased marker expression elicited by glial conditioned media. In fact, the combination of ghrelin and conditioned media consistently induced a further increase of PGP9.5 and MAP2 expression, especially after 7 days of treatment. The combination of ghrelin with SC-CM produced the most evident effects. Weak or no modifications were found on conditioned medium-induced GFAP increases. Observations on the ghrelin receptor indicate that its expression in control ASCs, virtually unchanged by the addition of only ghrelin, was considerably increased by CM treatment. These increases were enhanced by combining ghrelin and CM treatment, especially at 7 days. Overall, it can be assumed that ghrelin favors a neuronal rather than a glial ASC differentiation.

## Introduction

Experimental data indicate that Ghrelin peptide may play a role in multiple functions of the central nervous system. (Ferrini et al. 2009; Russo et al. 2018). It influences several neurological processes, such as learning and memory, stress response, sleep-awake cycle and behavior (Meyer et al. 2014; Jiao et al. 2017). Ghrelin also promotes neurogenesis (Sato et al. 2006). For example, its contribution has been reported in recovery and synaptic formation at the hippocampal level (Berrout and Isokawa 2012; Stoyanova et al. 2013; Kent et al. 2015). Moreover, emerging evidence indicates that ghrelin can also stimulate the proliferation, differentiation and migration of neural stem/progenitor cells (Xu et al. 2008).

The aim of this work was to test whether ghrelin affects Mesenchymal stem cell (MSC) neural differentiation. In fact, it has been demonstrated that MSCs can differentiate not only into mesenchymal cells, such as adipocytes, myocytes, osteocytes and chondrocytes (Maccarinelli et al. 2005; Kern et al. 2006; Lo Furno et al. 2013a; Naji et al. 2019), but also transdifferentiate into neural elements (Zhang et al. 2012; Goudarzi et al. 2018).

In previous studies, a neural differentiation of adipose-derived MSCs (ASCs) was achieved by their growth in a conditioned medium (CM) obtained from cultures of Olfactory Ensheathing cells (OECs) or Schwann cells (SCs) (Lo Furno et al. 2018a). OECs are specialized glial cells of the mammalian olfactory system characterized by continuous neurogenesis; they also support the growth of new olfactory receptor neurons (Pellitteri et al. 2010). OECs are found both outside and inside the central nervous system (Gómez et al. 2018) and, in physiological conditions, they drive the non-myelinated axons of receptor neurons towards the olfactory bulb by ensheathing nerve fibers and promoting their growth (Nazareth et al. 2015). SCs are located in the peripheral nervous system, where they myelinate large-diameter axons and provide trophic support for motor and sensory fibers. They contribute to the composition of the extracellular matrix, crucial for neuronal survival and axonal growth. SCs also contribute to axon restoration after injury (Golden et al. 2007). Both OECs and SCs originate from the neural crest (Barton et al. 2017) and produce numerous adhesion molecules, cytokines and neurotrophic factors, such as Brain-Derived Neurotrophic Factor (BDNF), Glial cell-Derived Neurotrophic Factor (GDNF), Nerve Growth Factor (NGF) and Neurotrophins.

In this investigation, ASC neural differentiation induced by OEC-CM or SC-CM was tested in combination with ghrelin added to the culture medium, to evaluate possible synergic or antagonist interactions. These combined effects were then compared to reference cultures consisting of ASCs cultured in OEC-CM or SC-CM, control ASCs grown in their basal medium, and control ASCs where ghrelin alone was added. ASC neural differentiation was assessed by evaluating the expression of typical neuronal markers such as Protein Gene Product 9.5 (PGP9.5) and Microtubule Associated Protein 2 (MAP2), as well as Glial Fibrillary Acid Protein (GFAP), indicative of a glial phenotype. Since interactions between ghrelin and its receptor have been recognized to be responsible for cell proliferation, differentiation and migration (Jiao et al. 2017), another set of experiments was devoted to explore the presence and possible modifications of the ghrelin receptor (GHSR-1a). In these experiments, both native and differently treated ASCs were investigated. Immunocytochemistry and flow cytometry were used to evaluate the presence and modifications of neural markers and GHSR-1a.

## Materials and methods

### Conditioned medium preparation

Conditioned media were obtained from glial cells of 2‐day old rat pups following experimental procedures carried out according to the Italian Guidelines for Animal Care (D. Lgs 26/2014), and the European Communities Council Directives (2010/63/EU). Protocols were approved by the ethics committee of the University of Catania (Organismo Preposto al Benessere Animale, OPBA; Authorization n. 174/2017-PR). All efforts were made to minimize animal suffering and to reduce the number of animals used.

#### OEC cultures and preparation of OEC-CM

Following procedures previously described (Pellitteri et al. 2009), olfactory bulbs were removed and OECs were isolated. Briefly, after removal, the bulbs were dissected (+ 4 °C) in Leibowitz l‐15 cold medium (Sigma–Aldrich, Milan, Italy). They were digested in Minimum Essential Medium‐H (MEM‐H, Sigma–Aldrich) containing collagenase (Invitrogen, Milan, Italy) and trypsin (Sigma–Aldrich) before adding Dulbecco's Modified Eagle's Medium (DMEM, Sigma–Aldrich) supplemented with 10% Fetal Bovine Serum (FBS, Sigma–Aldrich) to stop enzymatic activity. OECs were finally plated in flasks and kept in DMEM/FBS supplemented with cytosine arabinoside (10^−5^ M) as the antimitotic agent to reduce fibroblast proliferation. Subsequently, OECs were cultured at 37 °C in DMEM/FBS with the addition of 1% penicillin/streptomycin. 24–48 h after reaching confluence, OEC‐CM was collected, filtered to remove debris and detached cells and stored at − 20 °C before further use. OECs were identified by immunostaining for S-100.

#### Schwann cell cultures and preparation of SC‐CM

Schwann cells were harvested from sciatic nerves, which were exposed, removed, and kept in DMEM with the addition of 1% penicillin/streptomycin (Pellitteri et al. 2006). They were then digested in DMEM containing 0.1% collagenase and 2.5% trypsin, mechanically dissociated by trituration and filtered through a 150 µm nylon mesh. The day after seeding, the antimitotic agent cytosine arabinoside (10^−5^ M) was added to reduce fibroblast proliferation. Finally, SCs were resuspended in fresh medium and plated on 25 cm^2^ flasks. 24–48 h after reaching confluence, SC‐CM was collected, filtered to remove debris and detached cells, aliquoted and stored at − 20° C before further use. SCs were identified by immunostaining for S-100.

### ASC cultures

ASCs were isolated from adipose tissue, which was harvested from four healthy (32–38 years old) female donors undergoing liposuction procedures at the Cannizzaro Hospital, Catania (Italy). The donors were non‐smokers and occasionally took non‐steroidal anti‐inflammatory drugs. Lipoaspirate was obtained after donors signed a written informed consent to use the lipoaspirate for experimental procedures, which were carried out in accordance with the Declaration of Helsinki. The protocol was approved by the local ethics committee (Comitato etico Catania1; Authorization n. 155/2018/PO).

The raw lipoaspirate (50–100 ml) was washed with sterile phosphate‐buffered saline (PBS; Invitrogen) to remove red blood cells and debris, and incubated for 3 h at 37 °C with DMEM containing 0.075% of type I collagenase (Invitrogen). After inactivation of collagenase by adding an equal volume of DMEM/FBS, the lipoaspirate was centrifuged at 1200 rpm for 10 min. Pellets were then resuspended in PBS, and cells were filtered through a 100‐μm nylon cell strainer (Falcon BD Biosciences, Milan, Italy). Following a final centrifugation/resuspension cycle, cells were plated in T75 culture flasks (Falcon BD Biosciences) with DMEM/FBS, 1% penicillin/streptomycin, 1% MSC growth supplement (MSCGS; ScienCell Research Laboratories, Milan, Italy). After 24 h of incubation at 37 °C with 5% CO_2_, non‐adherent cells were removed by replacing the culture medium. After reaching confluence (about 80% of total flask surface), all cultures were trypsinized and cells were cultured for 2–3 passages before the subsequent procedures.

Some ASC samples were tested for their MSC nature, according to a protocol previously described (Lo Furno et al. 2018b). After 3 days from seeding, immunostaining for typical MSC markers (CD44, CD73, CD90, and CD105) was verified, as well as their immunonegativity for typical hematopoietic stem cell markers (CD14, CD34, and CD45). For the present investigation, six groups of ASC cultures were prepared. One group served as control, consisting of ASCs kept in basal growth medium (ASC); ASCs of the second group were cultured in basal growth medium with the addition of 2 µM ghrelin (ASC + Ghre); ASCs of the third group were cultured in OEC‐CM (ASC/OEC-CM); ASCs of the fourth group were cultured in OEC‐CM, in which ghrelin was added (ASC/OEC-CM + Ghre); ASCs of the fifth group were cultured in SC‐CM (ASC/SC-CM); and ASCs of the sixth group were cultured in SC‐CM in which ghrelin was added (ASC/SC-CM + Ghre). From each group, some samples were stopped at 1 day; other samples were processed after 7 days of culture. At each time point, fluorescence immunocytochemistry and flow cytometry procedures were carried out for signal detection.

### Flow cytometry

After trypsinization, cells of each sample were analyzed by flow cytometry. They were fixed with 2% paraformaldehyde in PBS for 20 min at 4 °C and permeabilized with 1% Triton for 5 min at 4 °C. After blocking nonspecific sites by BSA treatment (1% in PBS for 30 min), cells were incubated (60 min at room temperature) with primary antibodies: rabbit anti‐PGP9.5 (1:100; Novus Biologicals, Milan, Italy; NBP1-96612), mouse anti‐MAP2 (1:100; Covance; SMI-52), and mouse anti‐GFAP (1:100; Novus; NB120-10062). Finally, cells were incubated (60 min at room temperature in the dark) with goat anti‐mouse or goat anti‐rabbit secondary antibodies conjugated with fluorescein (FITC; 1:200; Abcam). Quantitative data were gathered using a Navios flow cytometer (Beckman Coulter, Milano; Italy). Samples were excited at 488 nm and fluorescence was monitored at 525 nm. Mean Fluorescence Intensity (MFI) values were calculated and recorded automatically. Kaluza Analysis Flow Cytometry Software was used for data processing.

### Immunofluorescence

Immunocytochemical staining was carried out following a protocol previously described (Lo Furno et al. 2013b). Briefly, cells were washed with PBS, fixed with 4% paraformaldehyde and incubated for 30 min with a 5% solution of normal goat serum (Sigma–Aldrich) in PBS containing 0.1% Triton (Sigma–Aldrich). They were then exposed overnight at 4 °C to primary antibodies: rabbit anti‐PGP9.5 (1:100; Novus Biologicals, Milan, Italy), mouse anti‐MAP2 (1:100; Covance), mouse anti‐GFAP (1:100; Novus), and rabbit anti‐GHSR-1a (1:100; Santa Cruz Biotechnology; sc-374515). The following day, cells were washed with PBS and incubated for 60 min at room temperature with secondary antibodies conjugated to different fluorochromes: FITC‐conjugated goat anti‐rabbit (Abcam) and/or Cy3‐conjugated goat anti‐mouse (Abcam). Finally, DAPI was applied for 10 min to identify cell nuclei. Some further samples were used to verify the specificity of immunostaining by omitting the primary antibody. Immunofluorescence was detected using a Leica DMRB Fluorescence Microscope equipped with the following Filter Cubes: Leica Filter Cube A (UV Excitation, P/N 513804); Leica Filter Cube I3 (Blue Excitation, P/N 513808); Leica Filter Cube N2.1 (Green Excitation, P/N 513812). A 40 × oil objective was normally used (Leica PL FLUOTAR, 40X/1.00–0.50 OIL, ∞/-/C). Digital images were acquired by a computer-assisted digital camera (Leica DFC 320, 3.3 Megapixel; Software: Leica Application Suite 2.8.1).

Immunostaining quantification was carried out through the FIJI-ImageJ measure tool (NIH, Bethesda, MD, USA). From each group, at least three samples were examined at each time point. From each sample, three digital photomicrographs were randomly selected. From each photomicrograph, up to seven immunofluorescent cells were analyzed. Values were derived from the average grayscale intensity. The integrated density, the cell area and the mean fluorescence of the selected cells were estimated. Three replicate measurements were performed for each capture region. The same procedure was applied to three different background areas, around the selected cell. Then, the Corrected Total Cell Fluorescence (CTCF) was calculated using the following equation: CTCF = integrated density—(cell area × background mean fluorescence).

### Statistical analysis

Statistical analysis was performed using GraphPad Prism 7.0 (GraphPad Software, La Jolla, CA, USA). For each experimental condition, CTFC values are reported as mean ± SD. Differences between samples were assessed using one-way analysis of variance (one-way ANOVA) followed by post hoc Holm–Sidak test. *p* values of 0.01 or less were considered statistically significant.

## Results

The stem cell profile of ASCs was verified by immunocytochemistry and flow cytometry. In accordance with previous studies (Lo Furno et al. 2018b), virtually all cells (98% or above) were immunopositive for typical MSC markers (CD44, CD73, CD90, and CD105), whereas less than 1% were positive for typical hematopoietic stem cell markers (CD14, CD34, and CD45). As expected, cell density at 7 days increased considerably when compared with day 1, especially in controls. In the same period, also cell morphology was noticeably changed. At 7 days, much larger cell bodies were more frequently found.

### Neural marker expression in ASCs

Neural marker expression modifications were evaluated by analyzing data obtained by flow cytometry and immunostaining quantification. Results consistently show that only weak effects were induced on neural marker expression when ghrelin alone was added to ASC cultured in their basal medium; significant increases were observed when ASCs were cultured in glial CM; dynamic effects could be observed when ghrelin was added to ASCs cultured in glial CM.

**Fig. 1:**
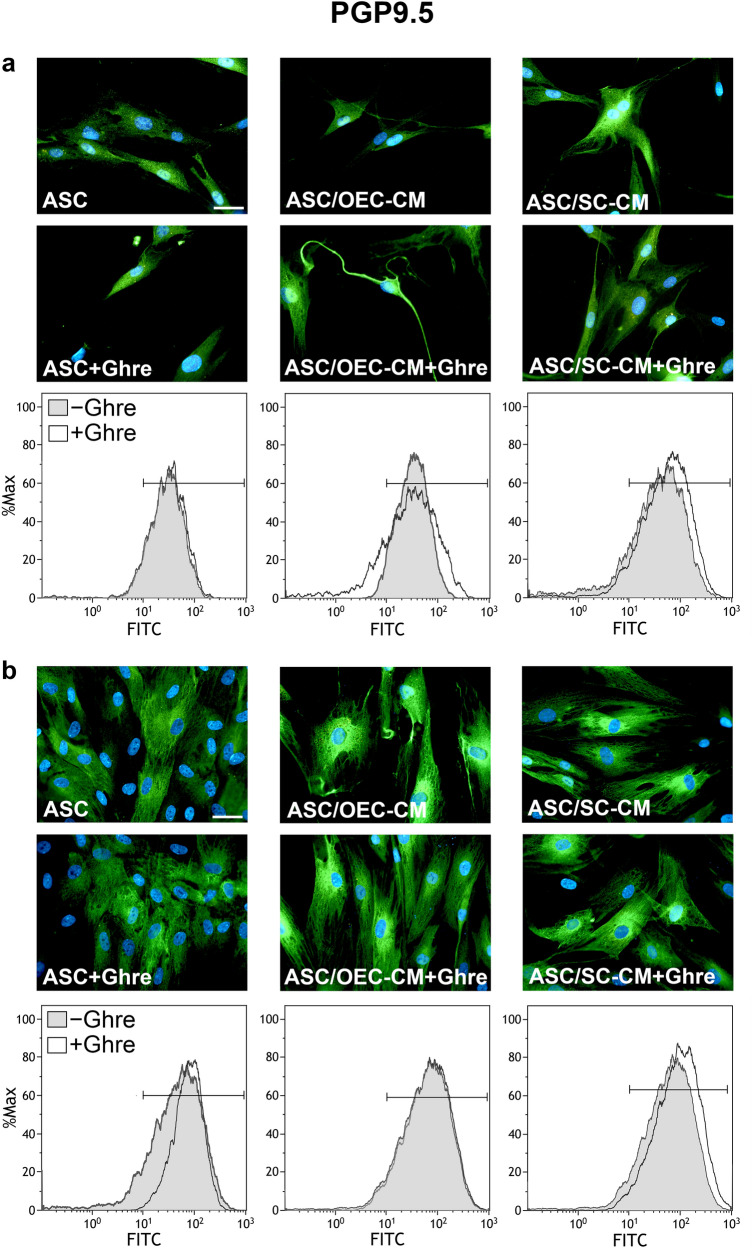
Modifications of PGP9.5 expression at 1 and 7 days of culture in ASCs undergoing different treatments. In panel **a** (1 day) and **b** (7 days), PGP9.5 immunoreactivity is shown in different groups of ASCs: ASC cultures in the basal medium (ASC); ASC cultures in OEC-CM (ASC/OEC-CM) or SC-CM (ASC/SC-CM); ASC cultures where ghrelin was added to the basal medium (ASC + Ghre), to OEC-CM (ASC/OEC-CM + Ghre) or to SC-CM (ASC/SC-CM + Ghre). In each flow cytometry plot, data obtained from each culture condition, with or without ghrelin, are merged. Histograms show cell frequency distribution of each population, according to FITC fluorescence intensity expressed in logarithmic scale. Histogram overlays are displayed as %Max, scaling each curve to the respective modal values = 100%. Scale bar: 50 μm

In agreement with previous data (Lo Furno et al. 2018a), the expression of PGP9.5 was higher in the ASCs cultured with OEC- or SC-CM when compared to controls (Figs. 1, 5a, Table 1), especially after 7 days of culture. A stronger effect seems to be exerted by SC-CM rather than OEC-CM treatment. The addition of ghrelin to SC-CM further increased PGP9.5 expression, whereas no appreciable modifications were observed when the peptide was added to OEC-CM. When ghrelin alone was added to ASCs cultured in basal medium, significant increases were observed only after 7 days of treatment. Immunofluorescence quantification data (Fig. 5a) and MFI values (Table 1) obtained from the different samples illustrate these modifications. Namely, the lowest MFI values were measured in control ASCs (34.79 and 69.36 at 1 and 7 days, respectively); the highest values referred to the population of ASCs undergoing combined ghrelin and SC-CM treatment (79.66 and 131.66 at 1 and 7 days, respectively). Altogether, data indicate that positive effects may be exerted by the presence of ghrelin on neuronal differentiation of ASCs. These influences are more evident after 7 days of treatment and significantly reinforce SC-CM-induced effects.

**Table1:** Effects of ghrelin and/or glial conditioned media on MFI modifications in ASCs at 1 and 7 days of culture

	PGP9.5		MAP2		GFAP	
	Day 1	Day 7	Day 1	Day 7	Day 1	Day 7
ASC	34.79	69.36	60.64	53.51	43.99	50.97
ASC + Ghre	37.73	89.13	56.39	52.13	50.01	51.01
ASC/OEC-CM	45.88	90.38	76.01	93.08	67.93	163.12
ASC/OEC-CM + Ghre	48.66	92.29	93.49	100.79	70.03	98.95
ASC/SC-CM	59.01	97.60	70.15	86.98	70.02	205.14
ASC/SC-CM + Ghre	79.66	131.66	89.32	122.95	72.10	167.89

**Fig. 2:**
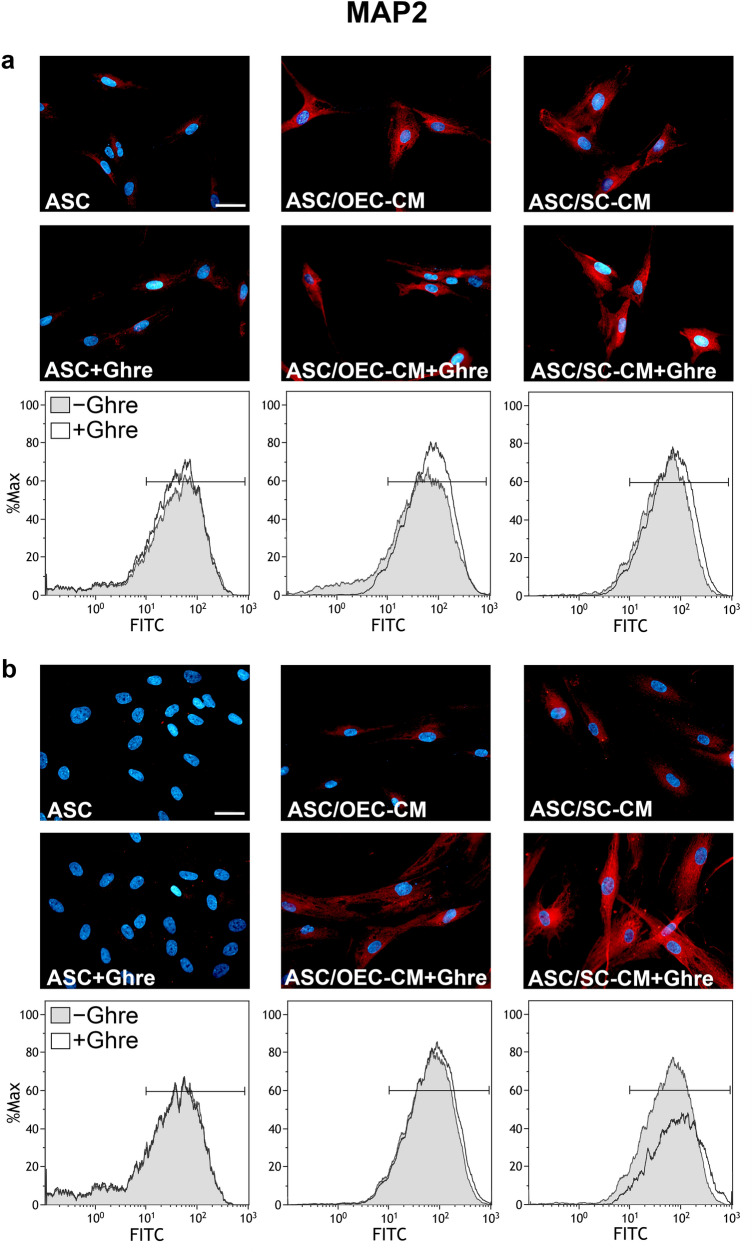
Modifications of MAP2 expression at 1 and 7 days of culture in ASCs undergoing different treatments. In panel **a** (1 day) and **b** (7 days), MAP2 immunoreactivity is shown in different groups of ASCs: ASC cultures in the basal medium (ASC); ASC cultures in OEC-CM (ASC/OEC-CM) or SC-CM (ASC/SC-CM); ASC cultures where ghrelin was added to the basal medium (ASC + Ghre), to OEC-CM (ASC/OEC-CM + Ghre) or to SC-CM (ASC/SC-CM + Ghre). In each flow cytometry plot, data obtained from each culture condition, with or without ghrelin, are merged. Histograms show cell frequency distribution of each population, according to FITC fluorescence intensity expressed in logarithmic scale. Histogram overlays are displayed as %Max, scaling each curve to the respective modal values = 100%. Scale bar: 50 μm

Regarding MAP2 expression, considerable increases were induced for glial CM treatment when compared with control ASCs, at both day 1 and 7 of culture (Figs. 2, 5b, Table 1). Immunofluorescence quantification data (Fig. 5b) show that ghrelin addition to OEC-CM further enhances MAP2 expression, although weakly, at both time points. Instead, the addition of ghrelin to SC-CM considerably increased MAP2 expression, but only after 7 days. No evident changes were detected by the addition of only ghrelin to ASCs cultured in basal medium. A similar trend can be recognized for MFI values showed in Table 1. Comparable low values were calculated for ASCs or ASC + Ghre (about 55), whereas the highest value referred to the population of ASCs undergoing combined ghrelin and SC-CM treatment at 7 days (122.95). It can be assumed that, although no effects seem to be exerted by ghrelin alone, a synergic influence occurs in combination with glial CM, especially for SC-CM at a longer time.

**Fig. 3:**
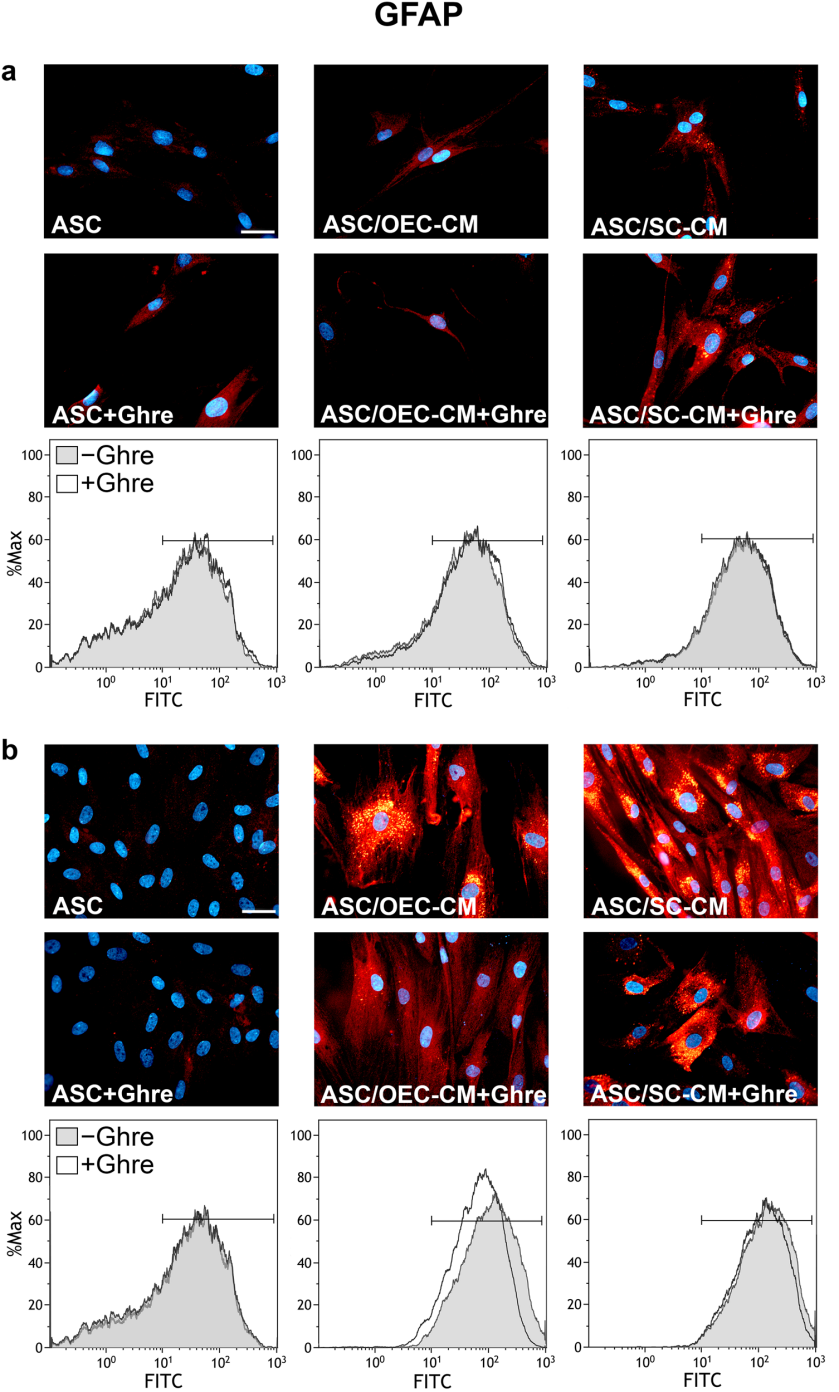
Modifications of GFAP expression at 1 and 7 days of culture in ASCs undergoing different treatments. In panel **a** (1 day) and **b** (7 days), GFAP immunoreactivity is shown in different groups of ASCs: ASC cultures in the basal medium (ASC); ASC cultures in OEC-CM (ASC/OEC-CM) or SC-CM (ASC/SC-CM); ASC cultures where ghrelin was added to the basal medium (ASC + Ghre), to OEC-CM (ASC/OEC-CM + Ghre) or to SC-CM (ASC/SC-CM + Ghre). In each flow cytometry plot, data obtained from each culture condition, with or without ghrelin, are merged. Histograms show cell frequency distribution of each population, according to FITC fluorescence intensity expressed in logarithmic scale. Histogram overlays are displayed as %Max, scaling each curve to the respective modal values = 100%. Scale bar: 50 μm

As expected, a robust increase of GFAP expression is easily appreciable at both 1 and 7 days (Figs. 3, 5c, Table 1) when ASCs are cultured in OEC-CM or, especially, SC-CM (Lo Furno et al. 2018a). However, as shown in Fig. 5c, these CM-induced increases were not greatly influenced by the addition of ghrelin; rather, unlike what was described for the other neural markers, slightly reduced glial-induced increases were observed. Again, only weak modifications were seen after the addition of ghrelin to ASCs cultured in basal medium. Table 1 shows that comparable low MFI values were measured for ASCs or ASC + Ghre (about 50), whereas the highest value referred to the population of ASCs cultured in SC-CM for 7 days (205.14). Also MFI values would suggest that ghrelin addition attenuates CM-induced increases; in particular, at day 7, MFI values decrease from 163.12 to 98.95 and from 205.14 to 167.89 for OEC-CM and SC-CM, respectively. From these results, it can be concluded that the presence of ghrelin would not support ASC differentiation toward a glial phenotype.

### Ghrelin receptor expression

**Fig. 4:**
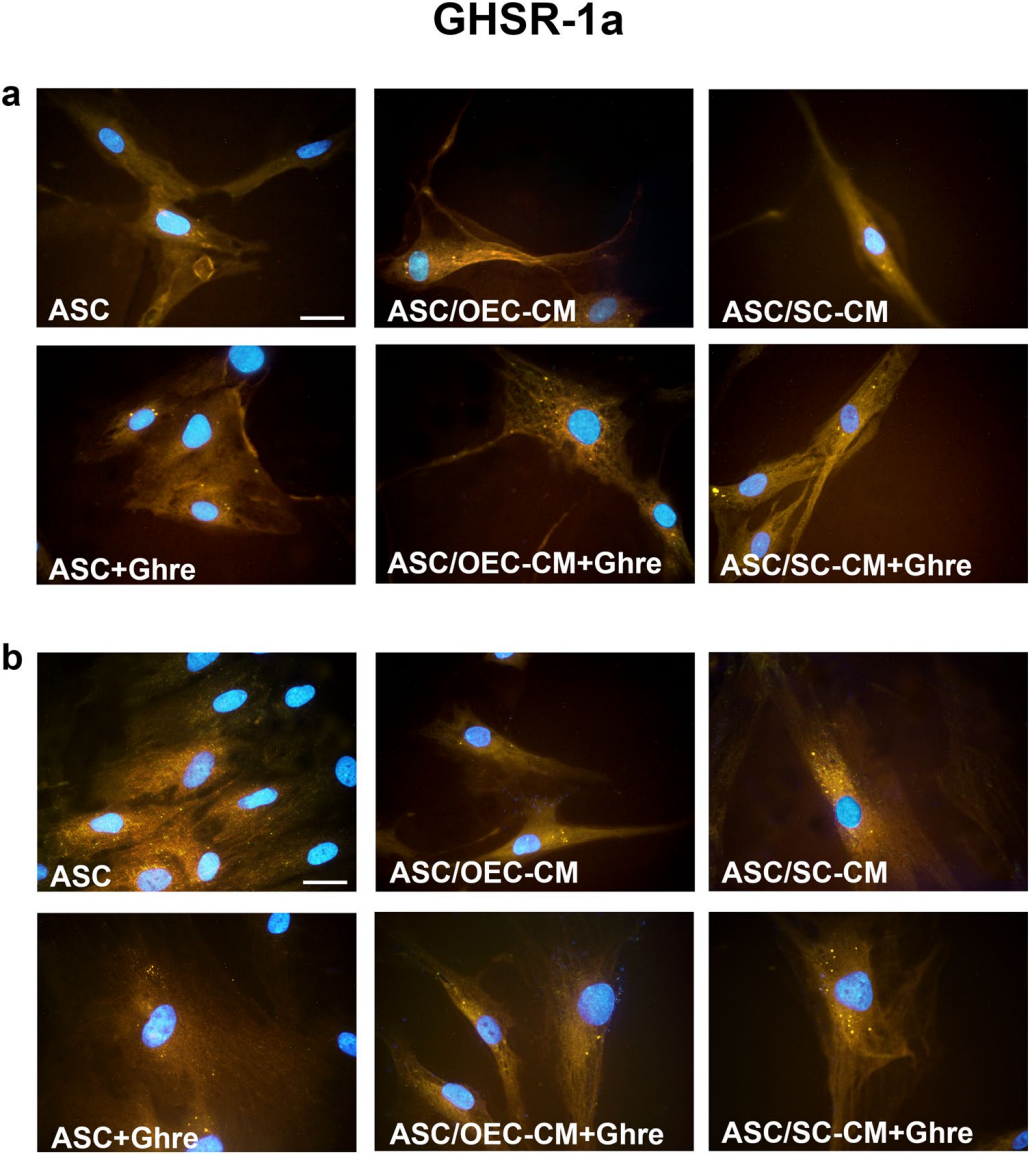
Modifications of ghrelin receptor (GHSR-1a) expression at 1 and 7 days of culture in ASCs undergoing different treatments. In panel **a** (1 day) and **b** (7 days), GHSR-1a immunoreactivity is shown in different groups of ASCs: ASC cultures in the basal medium (ASC); ASC cultures in OEC-CM (ASC/OEC-CM) or SC-CM (ASC/SC-CM); ASC cultures where ghrelin was added to the basal medium (ASC + Ghre), to OEC-CM (ASC/OEC-CM + Ghre) or to SC-CM (ASC/SC-CM + Ghre). Scale bar: 50 μm

**Fig. 5:**
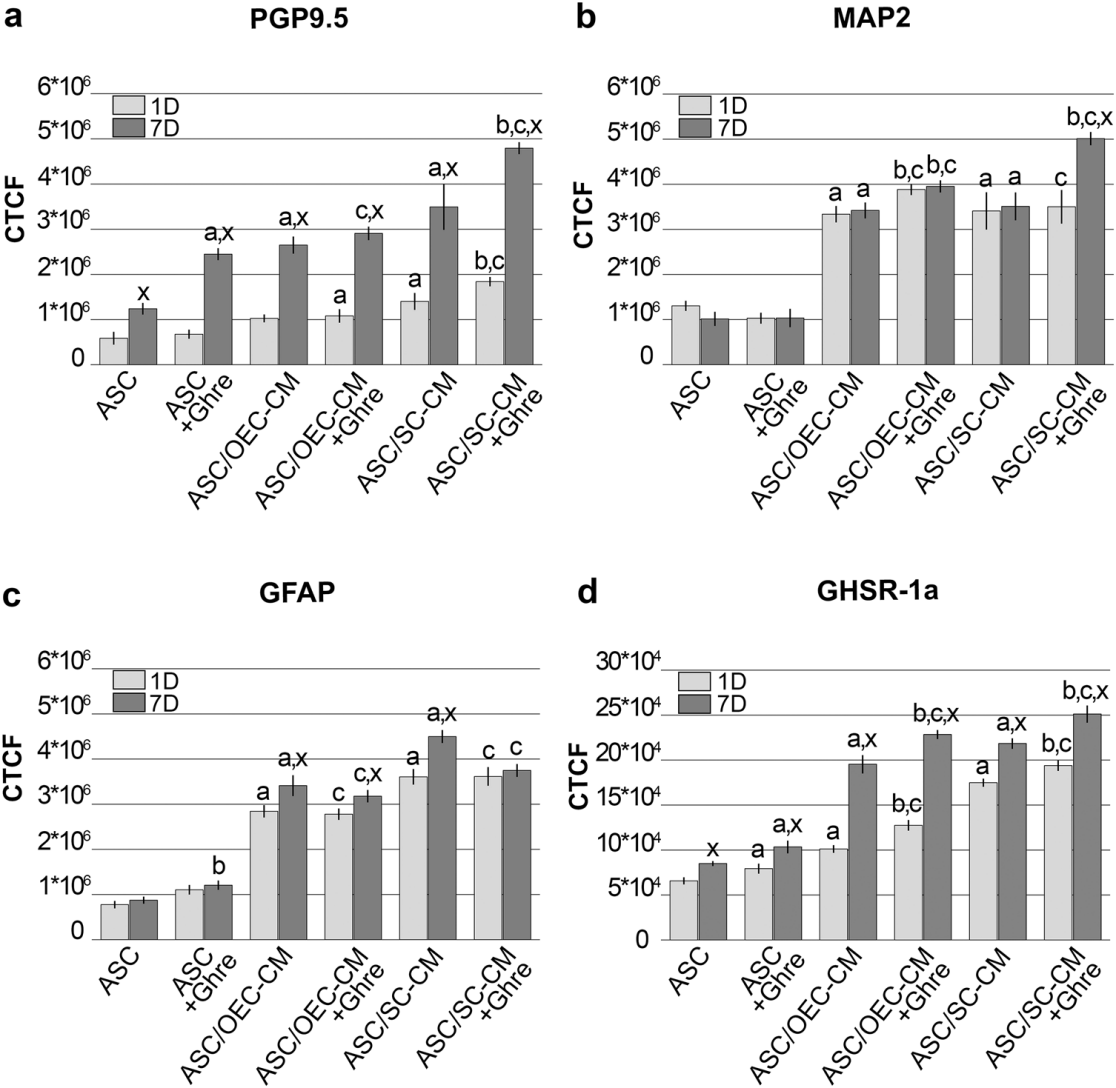
Histograms showing fluorescence quantification data for neural markers in each ASC group at day 1 and 7. **a** PGP9.5,** b** MAP2**, c** GFAP,** d** GHSR-1a. Bars represent CTCF mean value ± SD, obtained from at least three independent experiments. ^**a**^Significant differences (*P* < 0.01) vs ASCs; ^**b**^Significant differences (*P* < 0.01) between the CM + Ghre treatment and the CM treatment alone; ^**c**^Significant differences (*P* < 0.01) between CM + Ghre treatment and ASC + Ghre; ^**x**^Significant differences (*P* < 0.01) between data at 7 days and 1 day of culture

Experiments aimed at evaluating GHSR-1a expression on ASCs were carried out to explain ghrelin-induced influences. Immunocytochemistry (Fig. 4) and fluorescence quantification analysis (Fig. 5d) show that a basal GHSR-1a expression can be observed in control ASCs at both 1 and 7 days of detection. Instead, marked increases were induced by both CM treatments, especially on day 7. Such increases were weakly enhanced by the addition of ghrelin, once again after 7 days. Modest increases were detected by the addition of ghrelin alone.

## Discussion

Data obtained in the present study confirm previous results in which a successful neural differentiation was induced in ASCs by using conditioned media from OECs or SCs (Lo Furno et al. 2018a). By this strategy, also in the present work the expression of PGP9.5, MAP2 and GFAP was considerably more expressed. Overall, these findings fit quite well with data in the literature, in which effects on neurogenesis and neural differentiation processes are described for OECs and SCs. Glial effects are likely induced by the release of several growth factors such as NGF, BDNF, CNTF, and GDNF (Barton et al. 2017). Indeed, it is difficult to assess that a marker by itself is surely indicative of a given phenotype. For example, although at lower levels, PGP9.5 is also expressed in neuroendocrine cells as well as in gonads and in transdifferentiating epithelia (Rooman et al. 2002). However, according to the literature, PGP9.5 was originally detected as a “brain-specific protein”, accounting for about 5% of total neuronal proteins (Day and Thompson 2010; Bishop et al. 2016).

MAP2 may also be found in reactive astrocytes and oligodendrocyte precursors (Vouyiouklis and Brophy 1995; Charrigre-Bertrand et al. 1991), as well as in early neuronal and glial precursors (Rosser et al. 1997). However, it is generally considered as a neuronal, neurogenesis-associated, differentiation marker (Soltani et al. 2005; Korzhevskii et al. 2012; Kim et al. 2020). In fact, MAP2 is a neuron-specific cytoskeletal protein that stabilizes microtubules, especially in their dendritic arborization. Finally, although GFAP has been found in subpopulations of diseased neurons (Hol et al. 2003), or in multipotent neural stem cells of the adult mammalian brain (Mitteldorp et al. 2010), its expression is commonly considered specific of astrocytes (Zhang et al. 2019).

In the present investigation, both flow cytometry and fluorescence quantification data indicate that ghrelin addition induced dynamic effects on ASC neural differentiation. Indeed, very modest influences could be observed when the peptide alone was added to ASCs cultured in the basal medium. The only appreciable effects consisted of an increased PGP9.5 expression after 7 days of treatment. For this reason, similarly to what was described by Liu et al. (2020), we tested whether ghrelin could induce synergistic effects in combination with other neural-differentiation promoting protocols. Based on our previous findings (Lo Furno et al. 2018a), the combination with glial conditioned media was tested. By this combined strategy, more evident ghrelin-induced modifications were observed. Results show that modest influences were noticeable when ghrelin was added to OEC-CM. In this case, a weak increased expression of PGP9.5 and MAP2 was recognizable at both 1 and 7 days of treatment. Instead, more marked effects were detected when ghrelin was added to ASCs cultured in SC-CM, particularly after 7 days of treatment. On the other hand, an opposite tendency was revealed for GFAP expression modifications. In fact, by adding ghrelin to glial CM, reduced glial-induced increases were detected, especially after 7 days of treatment. In general, more marked increases of neuronal marker expression were observed when ghrelin was combined with SC-CM rather than with OEC-CM. Such differences might be due to different interactions between the peptide and glial-produced bioactive molecules. In this respect, it should be considered that many factors, even if produced by both OECs and SCs, can be released in different amounts, and might differently interact with ghrelin. Although the molecular interplay of these influences was beyond the purpose of this work, these interactions certainly deserve to be investigated.

In a recent work by Liu et al. (2020), ghrelin effects on ASC neural differentiation were investigated. Although rat ASCs and different neural induction strategies were used, results obtained largely match those here described. In fact, these authors report that the addition of ghrelin, in combination with a neurogenic culture medium, induced a dose-dependent increase of neural markers. Ghrelin effects were explained by an upregulation of the phosphorylation levels of AKT and mTOR, as well as the activation of the β-catenin signaling pathway. Different findings were however reported for GFAP, whose expression was found increased, although to a lesser extent than other neural markers. This discrepancy might be explained by taking into account the different experimental approaches, i.e. the different sources of ASCs and/or the different neural induction protocol. In our study, the use of glial conditioned media was preferred for ASC neural differentiation since this strategy would more closely mimic the physiological composition of a neural microenvironment in vivo.

Overall, also taking into account the limited observation period, ASCs would likely be at the early stages of neural differentiation, in which both neuronal and glial markers still coexist (Lo Furno et al. 2018b). However, based on these results, it is possible to conclude that, at least using the protocol adopted here, a neuronal rather than a glial commitment would be promoted. Otherwise, if a glial phenotype was favored, a further increase of GFAP expression should have been observed. This conclusion is in keeping with data reported by Gong et al. (2020), describing ghrelin effects on neural stem cells. In fact, whereas their differentiation towards dopaminergic neurons was enhanced through the activation of the Wnt/β‐catenin pathway, a significant decrease of GFAP‐positive cells was reported.

In later experimental steps, the expression of GHSR-1a was also investigated to explain ghrelin-induced effects. GHSR-1a is widely expressed in the hippocampus and in various other brain regions (Akalu et al. 2020). Notably, the expression of ghrelin and its receptor has been also demonstrated in OECs (Russo et al. 2020a, b). It is worth noting that also undifferentiated ASCs express appreciable levels of GHSR-1a. This basal expression was improved after the different ASC treatments. Analogously to what was found for neural marker modifications, only modest increases were observed by the addition of ghrelin alone. Instead, effects were considerably more pronounced when using glial conditioned media. For each group, increases were consistently more evident after 7 days of treatment. The increased expression of GHSR-1a after glial CM treatment might explain why ghrelin-induced effects were more evident in combination with CM, particularly after 7 days.

In conclusion, the present work confirms that ghrelin may play a role in neural differentiation processes. Although these effects might per se be of a modest entity, they would be reinforced by mutual interactions with growth factors or cytokines. As already suggested (Jiao et al. 2017), ghrelin neuroprotective and neurogenic roles may be usefully exploited in the development of therapeutic strategies for the treatment of neurodegenerative diseases. An in vivo administration would reveal whether ASCs predifferentiated following these combined strategies better work in the host tissue.

## Abbreviations

ASC: Adipose derived mesenchymal stem cells
ASC/OEC-CM: ASC cultures in OEC-CM
ASC/SC-CM: ASC cultures in SC-CM
CTCF: Corrected Total Cell Fluorescence
GFAP: Glial Fibrillary Acid Protein
Ghre: Ghrelin
GHSR-1a: Growth Hormone Secretagogue Receptor (Ghrelin Receptor)
MAP2: Microtubule associated protein 2
MFI: Mean fluorescence intensity
OEC-CM: Olfactory ensheathing cell- conditioned medium
PGP9.5: Protein gene product 9.5
SC-CM: Schwann cell-conditioned medium
